# Loss of Chemerin in Rhabdomyosarcoma Cells Polarizes Adjacent Monocytes to an Immunosuppressive Phenotype

**DOI:** 10.3390/biomedicines10102610

**Published:** 2022-10-18

**Authors:** Rui Sun, Jia Le Lin, Man Si Cheng, Kang Yi Lee, Thilo Spruss, Christa Buechler, Herbert Schwarz

**Affiliations:** 1 Department of Physiology, Yong Loo Lin School of Medicine, National University of Singapore, Singapore 117593, Singapore; 2 NUS Immunology Programme, Life Sciences Institute, National University of Singapore, Singapore 117456, Singapore; 3 NUSMED Immunology Translational Research Programme, National University of Singapore, Singapore 117456, Singapore; 4 Integrative Sciences and Engineering Programme, National University of Singapore, Singapore 117456, Singapore; 5 Veterinary Service, University of Regensburg, 93042 Regensburg, Germany; 6 Department of Internal Medicine I, University Hospital Regensburg, 93053 Regensburg, Germany

**Keywords:** chemerin, rhabdomyosarcoma, macrophage polarization

## Abstract

Chemerin is a multifunctional adipokine that regulates adipogenesis, insulin signaling and blood pressure and has thus a central function in metabolism. Mounting evidence confirmed a function of chemerin in various cancers. In this study, we investigated the role of chemerin in rhabdomyosarcoma (RMS), an aggressive soft tissue cancer that affects mainly children and young adults. We found chemerin expression in 93.8% (90 of 96) of RMS cases, with a range of 86.7–96.7% for the four RMS subgroups. While chemerin is uniformly expressed in normal skeletal muscle, its expression in RMS is patchy with interspersed areas that are devoid of chemerin. This variable chemerin expression is reflected by RMS cell lines as two of them (Rh41 and Rd18) were found to secrete chemerin while the two other ones (JR1 and RD) were negative. Deletion of chemerin in Rh41 and Rd18 cells did not alter their growth rate or morphology. We investigated the potential influence of chemerin on immune surveillance by coculturing parental and chemerin-deficient RMS cells with resting- or lipopolysaccharide (LPS)-activated human peripheral monocytes. The absence of chemerin in the RMS cells led to increased expression levels of the coinhibitory molecules PD-L1 and PD-L2 while levels of the costimulatory molecule CD86 were not changed. Further, the absence of chemerin enhanced the secretion of cytokines (IL-1β, IL-6, IL-10 and TNF) that have been shown to support RMS pathogenesis. These data indicate that the loss of chemerin expression by RMS cells repolarizes monocytes in the tumor microenvironment to supporting tumor progression.

## 1. Introduction

Chemerin, also known as retinoic acid receptor responder 2, is a multifunctional adipokine that is highly expressed by adipocytes and hepatocytes. Chemerin regulates adipogenesis, insulin signaling and blood pressure and thus has a central function in metabolism [1,2]. This adipokine is synthesized as pre-prochemerin. Following a 20-amino acid cleavage at the N-terminus, the 163 amino acid long human prochemerin is secreted into the bloodstream in an inactive form [3]. Depending on the biological process, a wide range of inflammatory proteases is available to cleave prochemerin at specific sites at the C-terminus, leading to different isoforms of active chemerin, including chemerin-156, chemerin-157 and chemerin-158 [4]. Chemerin exerts its functions by binding to chemokine-like receptor 1 (CMKLR1) and G protein-coupled receptor 1 (GPR1). C-C chemokine receptor-like 2 (CCRL2) is a non-signaling chemerin receptor, which functions to concentrate chemerin at specific sites [1,5].

There is ample evidence that chemerin influences cancer development. However, the exact role of chemerin in tumorigenesis is controversial since chemerin has been associated with pro-cancer, as well as anti-cancer, effects. The outcome may depend on its C-terminal processing, the local chemerin concentration, the specific cancer type and the tumor microenvironment [1,2,3,4,5].

Chemerin is a chemoattractant and recruits tumor-suppressive T cells, natural killer cells, dendritic cells and macrophages into the tumor microenvironment [3,6]. Activation of the tumor suppressor gene early growth response (EGR) 1 and of phosphatase and tensin homolog (PTEN) and the inactivation of the AKT, the mitogen-activated protein kinase (MAPK) p38 and the wingless-integrated (Wnt)/β-catenin pathways are further anti-tumor activities of chemerin [2,3].

On the other hand, chemerin can also promote tumor growth, for example, by enhancing angiogenesis or activating matrix metalloproteinases, AKT and p38, or by increasing the secretion of pro-inflammatory cytokines [2,3]. Inflammation, especially chronic inflammation, supports cancer development and progression [7,8]. 

Chemerin and its receptors are expressed in skeletal muscle [9,10,11]. Chemerin was reported to inhibit differentiation and to enhance cell proliferation of C2C12 myoblasts, which is mediated through the extracellular-signal-regulated kinase (ERK)-1/2 and mammalian target of rapamycin (mTOR) signaling pathways [12]. Impaired muscle cell differentiation and accelerated cell proliferation are characteristics of rhabdomyosarcoma (RMS), a malignancy of the muscle which most commonly develops in children [13,14,15]. 

RMS is classified according to histopathologic features. Embryonal RMS (ERMS) is the major subtype with a good prognosis and mostly occurs in children. Alveolar RMS (ARMS) is a more aggressive cancer type in young adults and adolescents. Pleomorphic RMS (PRMS) is an aggressive adult sarcoma. Spindle cell/sclerosing RMS (Sc-RMS) compromises 5–13% of RMS cases and is found in children and adults [13,14,16,17]. 

The aim of the current study was to evaluate the presence and role of chemerin in RMS. We found chemerin to be secreted by RMS cell lines and to be present in RMS biopsies.

## 2. Materials and Methods

### 2.1. Cell Lines

The human RMS cell lines Rd18, Rh41, RD and JR1 were kindly provided by Professor Reshma Taneja (National University of Singapore, Singapore) and Dr. Rossella Rota (Children’s Hospital Bambino Gesu, Rome, Italy). Parental and mutant RMS cells were cultured in RPMI 1640 (Gibco, Thermo Fisher Scientific, Waltham, MA, USA) supplemented with 10% fetal bovine serum (Gibco).

### 2.2. Chemerin Knock-Out

Stable knock-outs of chemerin were generated in the Rd18 and Rh41 cell lines by CRISPR/Cas9 with the gRNA sequence GACCAGTGTGGAGAGCGCCG cloned into the eSpCas9-2A-GFP plasmid (GenScript). Cells were transfected with TurboFect transfection reagent (Thermo Fisher Scientific, Waltham, MA, USA) and selected by several rounds of FACS for GFP-positive cells. Chemerin knock-out was confirmed by analysis of cell media using a chemerin ELISA.

### 2.3. RMS Cell–Monocyte Coculture

All blood samples were obtained from healthy donors at the Health Sciences Authority, Singapore, with approval from the Institutional Review Board, Singapore (IRB number: NUS-IRB-2020-67), in accordance with the guidelines of the Health Sciences Authority of Singapore. Peripheral blood mononuclear cells (PBMC) were prepared by Ficoll-Pague (GE Healthcare, Chicago, IL, USA) density gradient centrifugation. Monocytes were then isolated from PBMC using EasySep™ Human Monocyte Isolation Kit (Stemcell Technologies, Vancouver, BC, Canada). Isolated monocytes were stimulated with 100 ng/mL of LPS or treated with the respective solvent (phosphate-buffered saline) (Sigma-Aldrich, St. Louis, MO, USA) and added to tissue culture wells seeded with either parental or chemerin knock-out RMS cell line Rh41 (2 × 10^5^ cells/well) at a cell ratio of 3:1 for up to 2 days. Monocytes were then harvested for flow cytometry, and supernatants were stored at −20 °C for later ELISA measurements.

### 2.4. Immunohistochemistry

Tissue Arrays (SO2082b and HuFPT075) were ordered from US Biomax Inc (Rockville, MD, USA). Immunohistochemistry was performed by using the VEC-TASTAIN ABC KIT (Vector Laboratories, Biozol Diagnostica, Eching, Germany; order number: VEC-PK-4000) and the ImmPACT DAB peroxidase substrate (Vector Laboratories; order number: VEC-SK-4105). The IHC-plusTM RARRES2/Chemerin antibody (order number: LS-B13333) and the IHC-plusTM CHEMR23/CMKLR1 antibody (order number: LS-B12924) were from Biozol (Eching, Germany).

### 2.5. Enzyme-Linked Immunosorbent Assay (ELISA)

The concentrations of chemerin and cytokines in cell supernatants were quantified using DuoSet ELISA kits (R&D Systems, Minneapolis, MN, USA). All experiments were performed according to manufacturer’s protocol, and measurements were performed in duplicates. Readings were analyzed using Sunrise™ microplate reader (Tecan Group Ltd., Männedorf, Switzerland).

### 2.6. Flow Cytometry

Cells harvested after coculture were stained with PE-conjugated anti-human CD14 (Thermo Fisher Scientific), APC-conjugated anti-human PD-L1 (Biolegend, San Diego, CA, USA), FITC-conjugated anti-human PD-L2 (eBioscience, San Diego, CA, USA) and PerCP-eF710-conjugated anti-human CD86 (eBioscience) antibodies and LIVE/DEAD™ Fixable Near-IR Dead Cell Stain (Thermo Fisher Scientific). Results were obtained by Attune™ NxT Flow Cytometer (Thermo Fisher Scientific, MA, USA) and analyzed with FlowJo software, version 7.6.5 (FlowJo, LLC, Ashland, OR, USA).

### 2.7. Statistical Analysis

Statistical significance was determined by Student’s *t*-test using GraphPad Prism software version 8.0.1 (San Diego, CA, USA) and Pearson’s Chi-squared test. A *p* value of <0.05 was considered significant.

## 3. Results

### 3.1. Expression of Chemerin by RMS 

**Figure 1:**
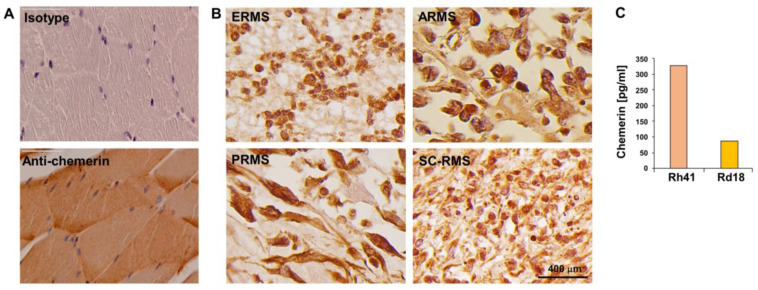
Chemerin expression by RMS. (**A**) Immunohistochemical stain of chemerin on normal skeletal muscle. Size bars represent 50 μm. (**B**) Immunohistochemical stain of chemerin on an RMS tissue microarray. Shown are 4 cores representing the 4 subtypes of RMS. ERMS (core F10, female, 42 years of age), ARMS (core L4, female 32 years of age), PRMS (core E11, male 67 years of age), SC-RMS (core A10, male 44 years of age). Size bars represent 400 μm. (**C**) Cells were cultured at 5 × 10^5^/mL for 24 h, and chemerin concentration in supernatant was determined by ELISA.

Normal muscle expresses chemerin, with staining being present throughout the tissue and in virtually all cells (Figure 1A). 

In order to evaluate chemerin expression in RMS, we stained a tissue microarray with 192 cores of 96 RMS patients (two cores per patient). Eleven cores had to be excluded due to loss during the staining procedure. In the remaining 181 cores, chemerin could be detected in 163, i.e., 90.1% of the cores (Figure 1B). Chemerin was present in all four subtypes of RMS; the vast majority (n = 90, 93.8%) of RMS cases was chemerin-positive, and in only six (6.25%), no chemerin could be detected (Table 1). These high numbers are not surprising, considering that healthy muscle uniformly expresses chemerin (Figure 1A). 

However, chemerin expression in RMS is patchy and not as homogeneous as in normal muscle (Figure 1B), and there are areas that are devoid of chemerin staining. The change in RMS versus healthy muscle consists in some cells having lost chemerin expression. We could not detect any significant associations between chemerin positivity and tumor stage (Table 2), gender (Table 3) or age of the patients (Table 4). Additional information on the patient population is shown in Supplementary Table S1.

Investigating four rhabdomyosarcoma cell lines, we found two of them, Rh41 and Rd18, to secrete chemerin while the two other cell lines, JR1 and RD, were negative (Figure 1C). Therefore, these cell lines seem to reflect the in vivo situation with varying chemerin expression in the RMS biopsies (Figure 1B). Stimulation of the cells with TNF, a proinflammatory cytokine, did not change chemerin secretion in Rh41 cells (not shown). 

### 3.2. Function of Chemerin in RMS

**Figure 2:**
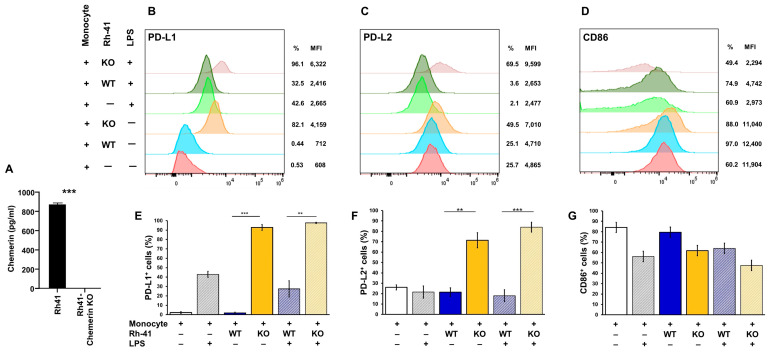
Function of chemerin in RMS. (**A**) Chemerin secretion in parental and chemerin KO Rh41 cells. (**B**–**G**) Influence of chemerin loss on cocultured monocytes. A total of 6 × 10^5^ monocytes, resting or activated with 100 ng/mL LPS, were cocultured with 2 × 10^5^ parental (WT) or chemerin-deficient (KO) Rh41 cells for 48 h. (**B**–**D**) Expression of PD-L1, PD-L2 and CD86 was analyzed by flow cytometry. (**E**) % PD-L1 positive cells. (**F**) % PD-L2 positive cells. (**G**) % CD86 positive cells. Depicted are means ± standard error of measurements of monocytes from 4 different donors. ** *p* < 0.01, *** *p* < 0.001, as determined by paired Student’s *t*-test.

Addressing the influence of chemerin secretion on RMS cells, we deleted chemerin by CRISPR-Cas9 in Rh41 and Rd18 cells (Figure 2A). Deletion of chemerin had no influence on the growth rate and morphology of the cells (not shown). In order to investigate the potential effect of chemerin on immune surveillance, we cocultured parental and chemerin-deficient RMS cells with primary monocytes, resting and after activation by LPS. Deletion of chemerin in the Rh41 cells causes a significant upregulation of the levels of the immunoinhibitory proteins PD-L1 and PD-L2, while levels of the immunostimulatory protein CD86 were unchanged (Figure 2B–G).

**Figure 3:**
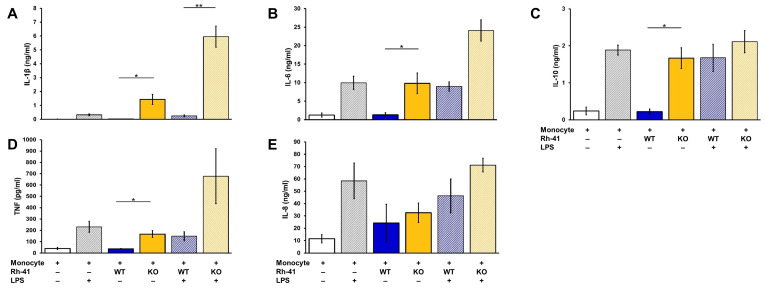
Chemerin regulates cytokine secretion in RMS cell monocyte cocultures. A total of 6 × 10^5^ monocytes, resting or activated with 100 ng/mL LPS, were cocultured with 2 × 10^5^ parental (WT) or chemerin-deficient (KO) Rh41 cells. After 48 h levels of IL-1beta (**A**), IL-6 (**B**), IL-10 (**C**), TNF (**D**) and IL-8 (**E**) in coculture, supernatants were determined by ELISA. Depicted are means ± standard error of measurements of monocytes from 4 different donors. * *p* < 0.05, ** *p* < 0.01, as determined by paired Student’s *t*-test.

Similarly, the secretion of the cytokine IL-1β was enhanced in resting and activated monocytes (Figure 3A) while secretion of IL-6, IL-10 and TNF was enhanced only in resting monocytes (Figure 3B–D). IL-8 secretion was also enhanced but to a lesser extent, and the change was not significant (Figure 3E).

## 4. Discussion

In this study, we addressed the role of chemerin in RMS which is to our knowledge the first of its kind. Only two of four tested RMS cell lines secrete chemerin. This fits the immunohistochemical data which show chemerin expression in many but not all cells of this cancer type. It also poses the question of whether chemerin is deleted in RMS or overexpressed. However, since chemerin is expressed by healthy muscle, it is more likely that some RMS cells lose chemerin during malignant transformation.

A significant association between chemerin positivity and the RMS tumor stage was not observed. Considering that there are four different types of RMS and that the prognosis varies depending on the location of the tumor, the number of patients was too small to identify such correlations [18].

Immunoediting of cancers, i.e., the changing of cancers in response to an anti-cancer immune response, is a well-known phenomenon and involves de novo or the increased expression of immunoinhibitory molecules and the loss of immunostimulatory ones [19]. We addressed the potential role of chemerin in monocyte activity in coculture experiments of chemerin-expressing and chemerin-deficient RMS cells with resting and LPS-preactivated primary human monocytes and found that the absence of chemerin leads to an increased expression of the coinhibitory molecules PD-L1 and PD-L2.

The PD-1–PD-L1 system is one of the most potent and best-characterized immune checkpoints. Expression of PD-L1 by tumors is a very effective and widespread strategy by various cancers to escape immune surveillance [20]. Accordingly, inhibition of PD-L1 or PD-1 has become an effective tool for treatment of many cancer types [21].

Moreover, it frequently is the macrophages in the tumor microenvironment, the tumor-associated macrophages (TAM), that express PD-L1 and PD-L2. It seems that loss of chemerin in RMS cells may promote or allow polarization of monocytes toward immunosuppressive TAM. About 6% of RMS cases were completely devoid of chemerin, and many RMS cases showed areas in which chemerin is lacking. It could be these areas, where macrophages express PD-L1 and PD-L2, that may suppress T-cell activity in the wider tumor microenvironment (TME). This increase in coinhibitory molecule expression, induced by the loss of chemerin, was not due to a general increase in cell surface marker expression since levels of the costimulatory molecule CD86 were not significantly changed.

Suppression of PD-L1 by recombinant, active chemerin was described in human sarcoma and prostate tumor cell lines [22]. Our current study shows that knock-out of chemerin in RMS cells causes an upregulation of PD-L1 and PD-L2 in monocytes. Chemerin thus may regulate PD-L1 expression of tumor cells and immune cells. It has to be noted that PD-L1 expression has been detected on TAM in RMS [23].

Interestingly, IL-1β, IL-6, IL-10 and TNF in cell media were significantly increased by the absence of chemerin. IL-10 is a potent immunoinhibitory cytokine that reduces expression of costimulatory and MHC molecules, and enhancement of IL-10 would act in concert with PD-L1 and PD-L2 and further reduce an anti-RMS T-cell response [24].

IL-1beta has many diverse functions and, aside from inducing innate immunity, it can support tumor development, e.g., by recruiting myeloid cells to the TME or by inhibiting T- and NK-cell activity [25,26].

Interaction of IL-6 with its receptor IL-6Rα and receptor subunit GP130 activates the PI3K/Akt, Ras/MEK/ERK and JAK/STAT3 pathways [27,28] in RMS. Among all these downstream effectors, STAT3 and Akt, in particular, mediate proliferation, apoptotic resistance and metastasis of RMS, suggesting the role of IL-6 in promoting the oncogenic transformation of RMS [29,30,31].

The absence of chemerin enabled an increase in TNF, a cytokine that despite its name, often contributes to tumor progression by contributing to a growth-conducive environment [32]. Further, TNF also induces the ectopic expression of the cytokine receptor CD137, which has been shown to facilitate the escape of RMS from immunosurveillance by activating a negative feedback mechanism which downregulates the immunostimulatory CD137L. Further, CD137 signaling in RMS cells induces the secretion of IL-6 and IL-8 [33].

An interesting question to be investigated in future research is whether the lack of chemerin in RMS cells induces other immunoinhibitory molecules aside from PD-L1 and PD-L2. Further, what is the mechanism leading to an upregulation of PD-L1 and PD-L2 levels and cytokine secretion caused by the absence of chemerin on RMS cells?

A limitation of our study is that we used an RMS cell line instead of primary RMS cells. However, primary RMS cells are difficult to obtain, genetically manipulate and keep in culture for extended periods of time. Another limitation is that the conclusions are based on the Rh41 cell line only. This is the case because we could not identify another RMS cell line with high chemerin expression.

In this study, we identified a tumor suppressive role of chemerin in RMS. In line with our findings, Pachynski and colleagues showed that chemerin suppressed melanoma by changing its immune environment. Depletion of natural killer (NK) cells abrogated the tumor-suppressive role of chemerin, illustrating that the anti-tumor effect of chemerin was mostly mediated through the attraction of NK cells. Chemerin did not have an effect on the proliferation of melanoma cells [6].

In hepatocellular carcinoma (HCC) cells, the tumor-suppressive effects of chemerin were related to a lower number of proangiogenic and immunosuppressive myeloid-derived suppressor cells and a higher number of cytotoxic T-cells [34]. It was moreover shown that hepatocyte-derived chemerin inhibits HCC cell migration and invasion [35]. Chemerin enhanced the expression of PTEN, thereby causing lower activation of AKT and subsequent suppression of migration, invasion and metastasis of the tumor cells [36].

However, overall, the role of chemerin in solid cancers is controversial since not all studies found a tumor-suppressive role. In esophageal carcinoma and ovarian cancer, chemerin is reported to exert tumor-promoting effects. Chemerin increased proliferation, migration and invasion of squamous cancer of the esophagus cells [37]. In ovarian cancer cells, chemerin upregulated PD-L1 thereby causing tumor cell proliferation and migration [38]. Elevated chemerin has also been linked to a worse prognosis in breast cancer [39] and in oral squamous cell carcinoma [40].

Moreover, the role of chemerin as a prognostic factor in solid tumors is uncertain and likely depends on the cancer type. This may partly be due to the fact that we do not know which chemerin isoform is produced by what cancers.

Similar to the identification of chemerin, other RMS-associated molecular changes have been recently identified that may become diagnostic parameters or even targets for therapy. E.g., a RAB3IP-HMGA2 fusion transcript is suspected to be a novel driver in a case of adult RMS [41,42]. Non-coding RNA is associated with the initiation, as well as progression, of RMS [43].

This study showed that chemerin is uniformly expressed in normal muscle, but its expression can be fully or partially lost in all four RMS subtypes. Loss of chemerin in RMS cells induces adjacent monocytes to express ligands for coinhibitory T-cell receptors and to secrete cytokines that support RMS progression.

## Figures and Tables

**Table 1:** Characteristics of patient tissues on the TMA. The column ‘Patients’ states the total number and the percentages of the total patient population for each RMS subtype. The column ‘Chemerin^+^ patients’ states the total number and the percentages of Chemerin^+^ patients per RMS subtype. There were 2 cores for each patient. The column ‘Chemerin^+^ cores’ states the total number and the percentages of Chemerin^+^ cores per RMS subtype. M, Males; F, Females.

	Patients	Sex		Chemerin^+^ Patients	Cores Excluded	Usable Cores	Chemerin^+^ Patients
		M	F				
ERMS	27 (28.1%)	17	10	25 (92.6%)	7	47	43 (91.5%)
ARMS	24 (25.0%)	12	12	23 (95.8%)	0	48	46 (95.8%)
PRMS	30 (31.3%)	19	11	29 (96.7%)	3	57	53 (93.0%)
SC-RMS	15 (15.6%)	13	2	13 (86.7%)	1	29	21 (72.4%)
Total	96 (100%)	61	35	90 (93.8%)	11	181	163 (90.1%)

**Table 2:** Patients and chemerin status by cancer stage. *p* = 0.421 by Chi-squared test. Evaluated were only patients with readable cores.

Stage	Chemerin^+^	Chemerin^−^	Total
IIA	4	1	5
IIB	9	0	9
III	73	4	77
IV	3	0	33
Total	89	5	

**Table 3:** Patients and chemerin status by gender. *p* = 0.413 by Chi-squared test. Evaluated were only patients with readable cores.

Gender	Chemerin^+^	Chemerin^−^	Total
Female	34	1	35
Male	55	4	59
Total	89	5	94

**Table 4:** Patients and chemerin status by age. *p* = 0.851 by Chi-squared test. Evaluated were only patients with readable cores.

Age	Chemerin^+^	Chemerin^−^	Total
1–10	7	0	7
11–20	13	1	14
21–30	14	0	14
31–40	16	2	18
41–50	20	1	21
51–60	7	0	7
61–70	5	1	6
71–80	3	0	3
81–90	3	0	3
91–100	1	0	1
Total	89	5	94

## Supplementary Materials

The following supporting information can be downloaded at: https://www.mdpi.com/article/10.3390/biomedicines10102610/s1, Table S1: Baseline characteristics of patients among different types of rhabdomyosarcoma. Numbers in brackets represent percentages of cases within each type of RMS.
