# Supplementary material for: Loss of Chemerin in Rhabdomyosarcoma Cells Polarizes Adjacent Monocytes to an Immunosuppressive Phenotype
Source: Biomedicines. 2022 Oct 18;10(10):2610. doi: 10.3390/biomedicines10102610 (PMC9599404; doi:10.3390/biomedicines10102610)
Supplement: Supplementary file 1 [file biomedicines-10-02610-s001.zip › biomedicines-1902366-supplementary.pdf]

Supplementary Table S1. Baseline characteristics of patients among different types of rhabdomyosarcoma. Numbers in brackets represent percentages of cases within each type of RMS.

		Alveolar RMS	Embryonal RMS	Pleomorphic RMS	Spindle cell RMS
**Total**		20	19	24	10
**Sex**					
	M	10 (50%)	11 (58%)	15 (63%)	9 (90%)
	F	10 (50%)	8 (42%)	9 (37%)	1 (90%)
**Age**		30 (10-81)	42 (1-82)	49 (8-91)	37 (10-64)
**Site of tumour**					
	Urogenital	3 (15%)	5 (26%)	6 (25%)	9 (90%)
	Retroperitoneum	1 (5%)	1 (5%)	3 (12.5%)	1 (10%)
	Limbs	13 (65%)	3 (16%)	12 (50%)	0 (0%)
	Head & Neck	0 (0%)	6 (32%)	0 (0%)	0 (0%)
	Others	3 (15%)	4 (21%)	3 (12.5%)	0 (0%)
